# Experiences of Mothers/Caregivers of Neonates Who Received Novel Positioning Along With Physiotherapy Interventions in the Intensive Care Unit: A Qualitative Study

**DOI:** 10.7759/cureus.89243

**Published:** 2025-08-02

**Authors:** Dhwani D Chanpura, Neha Mukkamala, Nalina Gupta

**Affiliations:** 1 Pediatric Physiotherapy, College of Physiotherapy, Sumandeep Vidyapeeth Deemed to be University, Vadodara, IND; 2 Musculoskeletal Physiotherapy, College of Physiotherapy, Sumandeep Vidyapeeth Deemed to be University, Vadodara, IND; 3 Neurological Physiotherapy and Community Rehabilitation, Maharishi Markandeshwar College of Physiotherapy and Rehabilitation, Maharishi Markandeshwar University, Sadopur, IND

**Keywords:** experiences, nicu, physiotherapy interventions, positioning, preterm neonates

## Abstract

This study aims to explore the caregiver empowerment regarding the novel device and physiotherapy interventions in the neonatal intensive care unit (NICU). Physiotherapeutic interventions in the NICU are among the earliest neurodevelopmental strategies provided to preterm infants. These include multisensory stimulations, along with flexed positioning that can support developmental progress. However, the emotional toll on mothers and caregivers during a neonate’s NICU admission, especially with added therapeutic procedures, remains a less explored domain. Gaining insight into their experiences is crucial for enhancing both developmental and emotional care practices. Ninety mothers/caregivers of preterm neonates (<37 gestational weeks), who were medically stable within the first 72 hours of life, had no congenital or neurological impairments, and received physiotherapy and positioning in a novel postural supporting device till admission to discharge, were included in this study. A descriptive qualitative design was employed. A structured, self-developed questionnaire was used for a brief interview of five to 10 minutes. Transcripts were thematically analyzed using content analysis. Five primary themes emerged: affective change; improvement in activity; improvement in participation; reduction in hospitalization; and empowerment in post-discharge care. The study highlights the emotional and developmental significance of physiotherapy interventions along with positioning for both neonates and their caregivers. Mothers experienced increased confidence, participation, and emotional support, underscoring the need for family-centered approaches in NICU physiotherapy.

## Introduction

In recent decades, advanced technological resources like mechanical ventilators and cardiorespiratory monitors have been available in neonatal intensive care units (NICUs), which have led to the survival of a large number of premature newborns. Preterm infants who do not receive proper interventions end up having long-term disabilities [1,2]. Among the multifaceted approaches to improving neonatal outcomes, physiotherapy, including interventions such as multisensory stimulation, respiratory facilitation, and therapeutic positioning, has emerged as a valuable adjunct to medical care in supporting neuro-motor development in the NICU [3].

Among the many physiotherapeutic strategies used in NICUs, therapeutic positioning has gained particular attention. These positioning techniques, often designed to support the baby’s posture to mimic the womb, help promote better musculoskeletal alignment and neuro-behavioral and postural development. These interventions have shown promise in enhancing both short- and long-term outcomes for neonates [4]. Much research has recognized the effects of these interventions, but fewer studies have examined how caregivers, especially mothers, experience and interpret these therapies while their babies are in intensive care [5]. Family-centered care (FCC) in the NICU is crucial for improving outcomes for both infants and their families. It emphasizes the active involvement of parents in their child's care, promoting bonding, reducing stress, and enhancing parenting confidence. This approach recognizes parents as essential members of the care team and aims to minimize the potential trauma of a NICU stay.

For mothers, having their newborn in the NICU can lead to feelings of fear, helplessness, guilt, and uncertainty. They feel unable to provide the hands-on care as they imagined during pregnancy. However, participating in positioning and during physiotherapy sessions, they felt empowered and developed a sense of connection, and were able to observe improvement in their baby’s development. This can play an important role in shaping a mother’s experience and emotional recovery during this difficult time [5-7].

While the importance of caregiver experiences in the NICU has been acknowledged in existing literature, a significant gap remains in understanding how novel positioning techniques combined with physiotherapy interventions affect these experiences, particularly from the perspective of mothers or primary caregivers of preterm neonates. Most studies to date focus either on general parental stress, developmental outcomes of the neonate, or the clinical efficacy of physiotherapy alone. This study uniquely explores caregivers’ experiences in a setting where a structured, novel positioning device was used alongside physiotherapy interventions, offering a holistic understanding of how such interventions impact caregiver perceptions, emotional adjustment, confidence in care, and perceived involvement in their infant’s developmental journey. These insights are crucial for designing family-centered NICU care. This study, therefore, aims to explore the experiences of mothers or caregivers whose newborns received both a novel positioning device and physiotherapy interventions in the NICU. Their reactions, perspectives, and emotions on novel positioning and neonatal physiotherapeutic interventions can be identified through in-depth interviews. These insights can help shape more empathetic, inclusive, and effective approaches to neonatal physiotherapy and care delivery in intensive care environments.

## Materials and methods

This study was approved by Sumandeep Vidyapeeth Institutional Ethics Committee (SVIEC/ON/PHYS/PhD/22012) and conducted in the Neonatal Intensive Care Unit of Dhiraj General Hospital, Gujarat, from June 2024 to January 2025. Written informed consent was obtained from the caregivers/guardians of all enrolled preterm neonates before commencement of the study. All individuals who were approached agreed to participate in the study, and none of the enrolled participants withdrew or dropped out during the course of the research.

A total of 90 primary caregivers of preterm neonates who received novel positioning and physiotherapy in the NICU were recruited using purposive sampling. Participants were selected based on their direct involvement in the infant’s care and willingness to participate, and were approached in person by a trained researcher once the neonates were clinically stable. The sample size was guided by qualitative research standards to ensure data saturation, thematic diversity, and credibility. Although saturation was reached around the 70th interview, all 90 interviews were completed to capture a broader range of experiences. The sampling strategy was reviewed and approved by an institutional biostatistician. Sampling bias was minimized by consistently applying inclusion criteria and recruiting at varied times to ensure representation across different caregiver backgrounds.

All the included preterm neonates ranged from 30 weeks 0/7 days to 36 weeks 6/7 days, all were medically stable in the first 72 hours of life, and referred for physiotherapy. Neonates were excluded if they required oxygen therapy or any form of respiratory support, such as mechanical ventilation, continuous positive airway pressure (CPAP), or high-flow nasal cannula, beyond 72 hours after birth, or any diagnosed neurological, musculoskeletal, genetic, chromosomal, or metabolic disorders.

**Figure 1:**
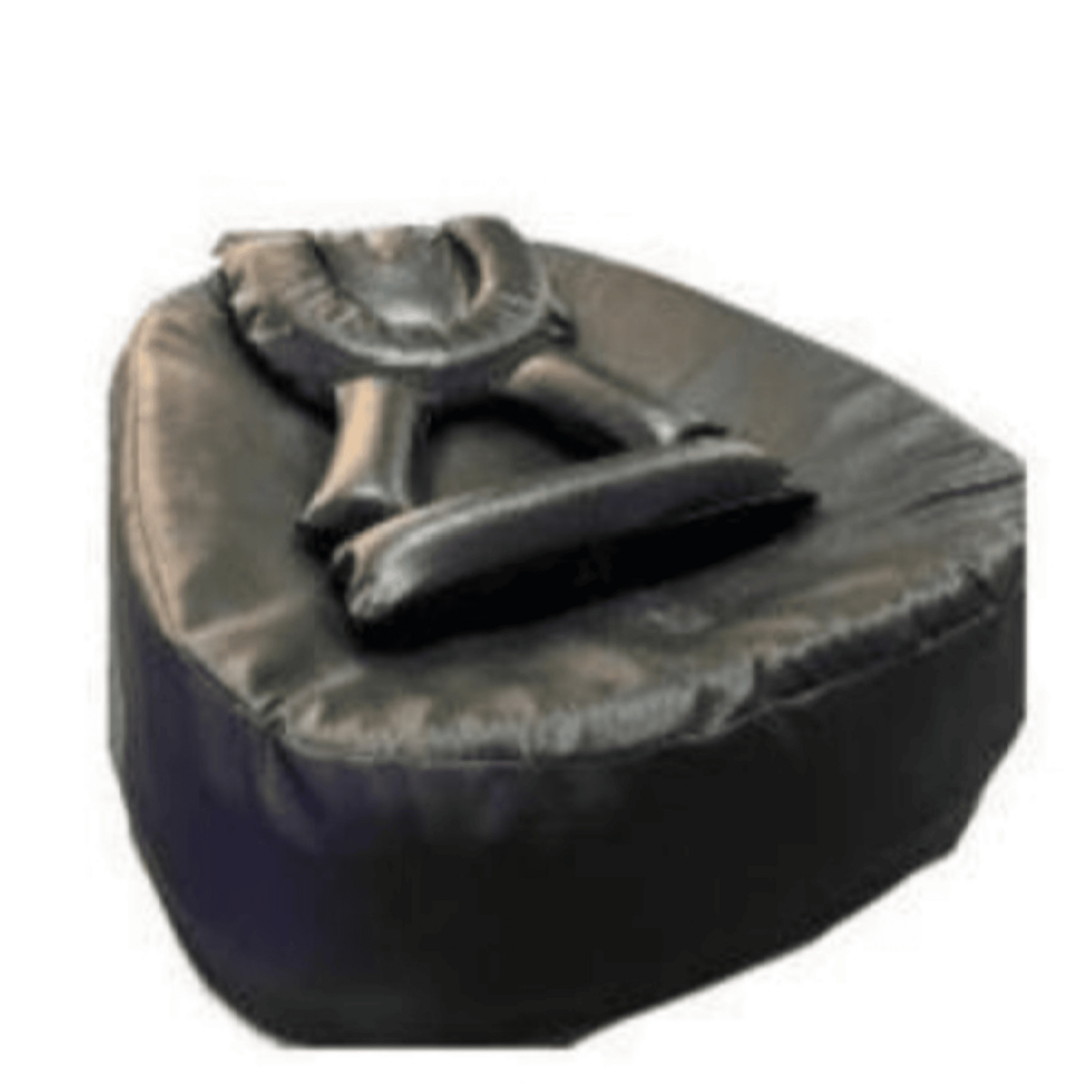
Postural supporting device. The novel “Postural Supporting Device” has been developed by the authors and published with Patent Design No: 403807-001.

All the included neonates received neonatal physiotherapy interventions for approximately 20 minutes daily and were positioned using a newly developed postural support device for 24 hours until discharge. This intervention (multisensory stimulation and oromotor stimulation) was based on the recommendations provided by the American Physical Therapy Association [8,9]. A novel postural supporting device was developed by the authors for preterm neonates in the NICU. The goal of the novel positioning was to mimic the flexed position, like the womb position. It is made from medical-grade memory foam and cotton with adjustable parts that support the head and neck in midline, shoulders in a protected position, along with the hip and knees in a flexed position. It was tested for safety, usability, hygiene, and ease of use for clinical integration. All infection control guidelines as per hospital policy were strictly followed. The positioning device was thoroughly disinfected between each use. Caregivers and healthcare staff adhered to standard hand hygiene. All physiotherapy sessions and novel positioning interventions were delivered by the same trained neonatal physiotherapist to maintain inter-rater and intra-provider consistency. This novel “Postural Supporting Device” has been developed by the authors and published with Patent Design No: 403807-001 (Figure 1).

Parents also participated in treatment sessions and were advised to perform these activities whenever they visited their baby in the NICU. The intervention was administered while the child was awake, preferably before the next scheduled feeding. It was discontinued if the child showed any signs of stress, such as fussing, crying, or sleeping. At the time of discharge from the hospital or during follow-up visits at the hospital, author DC, who is a neonatal physiotherapist working in the NICU for more than 10 years, conducted an in-depth interview using a self-made structured questionnaire about their experiences of novel positioning and neonatal physiotherapy. She was trained in qualitative interviewing techniques, including unbiased questioning and active listening, and conducted mock interviews under supervision to ensure calibration and consistency. To minimize bias, she is not involved in any of the interventions. The questionnaire underwent expert review for face and content validity by professionals in neonatal physiotherapy, neonatologists, neonatal nurses, and clinical psychologists. To ensure consistency, all interviews were conducted by a single trained interviewer, and thematic coding was performed independently by two researchers with consensus reconciliation.

Interviews were conducted in the participants’ regional languages and were professionally translated into English. Translation fidelity was ensured through back-translation and cross-checking by bilingual experts to maintain the accuracy and meaning of participants’ responses. Each interview session lasted approximately 10 minutes and involved oral responses to a structured questionnaire conducted in the caregiver’s regional language by a trained interviewer. It was a guided interview (Table 1).

**Table 1:** Questionnaire on experiences of mothers/caregivers of neonates who received physiotherapy intervention in the NICU. Dear mothers/caregivers, we would like to ask you certain questions about the changes in your neonates’ motor and behavioral responses after receiving physiotherapy intervention and positioning. If you observed any changes, please elaborate. We would be very grateful for your responses.

Sr. No.	Question
1	Do you feel any change in your neonates' position (posture)?
2	Do you feel any change in the way your neonate tries to look at you?
3	Do you feel any change in your neonate's response when you call/talk to him/her?
4	Do you feel any change in your neonate's alertness/awareness/attention?
5	Do you feel any change in your neonate's activity/energy levels in body/limb movements?
6	Do you feel any change in your neonate's milk-sucking response?
7	Do you feel any change in your neonate's feeding patterns?
8	Do you feel any change in your neonate's sleep patterns?
9	Do you feel any change in your neonate's weight?
10	Do you feel any change in your neonate's duration of hospital stay?
11	Do you feel confident/helpful in handling/managing your neonates at home?
12	Any other relevant information

## Results

Basic characteristics of the included preterm neonates are presented in Table 2.

**Table 2:** Demographic characteristic data (gestational age, gender, requirement of oxygen support, and hospital stay) of the included preterm newborns.

Characteristics	Category	Number (n)	Percentage (%)
Gestational age (weeks)	30-32 weeks	23	25.56
	32-34 weeks	46	51.11
	34-36 weeks	21	23.33
Gender	Male	52	57.78
	Female	38	42.22
Requirement of oxygen support	Yes	27	30
	No	63	70
Hospital stay (days)	Less than 7 days	67	74.45
	More than 7 days	23	25.56

Thematic analysis was conducted manually by two independent researchers to ensure reliability and reduce bias. Both coders initially reviewed transcripts independently and then met to discuss emerging codes and patterns. A coding framework was developed collaboratively, beginning with open coding and gradually refined through comparison and discussion. Codes were grouped into broader categories, and themes were defined based on recurring patterns across interviews. This process was iterative, involving regular back-and-forth adjustments to ensure consistency and clarity. Although no qualitative data analysis software was used, meticulous manual documentation supported the process. Consolidated Criteria for Reporting Qualitative Research (COREQ) guidelines were adhered to throughout the analysis.

Five main themes emerged from the qualitative analysis of mothers/caregivers' interviews (Table 3).

**Table 3:** Themes and parents’ perspectives.

Sr. No.	Theme	Generalized statements
1.	Affective change	At the time of admission, many caregivers experienced intense emotional distress and fear, often hesitating to interact with their neonates.
		Caregivers commonly felt helpless, observing their neonates in critical care settings, unsure of how to contribute.
		Over time, caregivers began to emotionally adapt to the NICU environment, although feelings of vulnerability persisted during the early days.
		Regular interaction with physiotherapists, particularly observing their calm and skilled handling of neonates, provided caregivers with emotional relief and reassurance.
		Observing positive changes in their infants during therapy sessions fostered hope and emotional comfort among caregivers.
2.	Improvement in activity	Caregivers observed improvements in their neonates’ activity levels, such as more frequent stretching and purposeful movements, following positioning and therapy.
		Neonates began responding more actively to sensory input, including touch and sound, indicating improved interaction and awareness.
		Caregivers noted visible changes in posture, including reduced stiffness and more relaxed body tone, after therapeutic interventions.
		Many neonates appeared more settled and emotionally regulated following therapy, reflecting better behavioral organization.
		Improvements in breathing patterns and coordinated limb movements were observed, reinforcing caregivers’ confidence in the therapeutic process.
		Physical and behavioral changes helped alleviate caregivers’ initial feelings of helplessness and increased their trust in the therapeutic outcomes.
3.	Improvement in participation	Caregivers initially engaged by observing physiotherapy sessions, gradually becoming familiar with therapeutic procedures.
		Over time, many caregivers transitioned from passive observation to active participation, contributing in therapy sessions under guidance.
		Direct involvement in therapeutic handling increased caregivers’ confidence in positioning and managing their neonates.
		Physiotherapists actively encouraged caregiver participation, creating a collaborative and supportive care environment.
		Being included in therapy sessions allowed caregivers to bond more closely with their infants, enhancing emotional connection.
		Active involvement counteracted feelings of helplessness or exclusion, making caregivers feel competent and empowered in the NICU.
4.	Reduction in hospitalization	Caregivers observed early developmental improvements that were associated with positive feedback from the medical team regarding potential early discharge.
		Many caregivers attributed their neonates' quicker recovery to the effects of physiotherapy and positioning interventions.
		Improvements in functions were noted by caregivers as contributing factors to medical stability and readiness for discharge.
		The transition toward discharge was reassuring for caregivers, reducing their stress and reinforcing their belief in the effectiveness of therapy.
		Many participants believed that positioning and physiotherapy played a key role in reducing the duration of hospitalization by enhancing the babies' development.
5.	Empowerment in post-discharge care	Caregivers reported increased confidence in managing their infant’s care at home due to the training and guidance received during physiotherapy sessions.
		Many caregivers continued to apply therapeutic techniques post discharge, demonstrating a sense of preparedness and skill retention.
		Involvement in NICU care and hands-on experience during therapy enhanced caregivers’ emotional readiness for the transition from hospital to home.
		The knowledge and skills acquired through active participation in therapy contributed to a sense of empowerment and self-efficacy in caregiving.
		Mothers felt they were well-equipped to support their infant’s development at home, reinforcing their role as primary caregivers beyond the NICU environment.

Among all mothers who had been involved in the care of neonates at the time of therapy sessions, they felt more confident and prepared. They felt empowered at the time of discharge and confident in caring for their neonates at home.

## Discussion

This qualitative study explored the experiences of mothers and caregivers whose neonates received novel positioning along with physiotherapy interventions in the NICU. The findings offer rich insights into the emotional and psychological transformations caregivers undergo when meaningfully involved in their infant’s therapeutic care. These experiences not only highlight the immediate developmental benefits for the neonates but also underscore the significant emotional reassurance and empowerment gained by the caregivers during a particularly vulnerable time.

Initially, many mothers described feelings of fear, helplessness, and uncertainty, which are well-documented reactions among parents of preterm neonates admitted to intensive care units [6,10]. The high-stress environment, combined with the fragile medical condition of their infants, contributed to a sense of emotional isolation and loss of control. However, a key turning point in their experience was the consistent presence and guidance of physiotherapists. As supported by previous literature, therapeutic engagement, when supported by trained professionals, can help parents transition from passive observers to active participants in their child’s care [6,10,11].

The physiotherapy sessions and novel positioning interventions served not only as clinical practices but also as gateways for mothers to establish a deeper emotional connection with their infants. Learning how to touch, observe, and interact with their babies under professional supervision helped many caregivers overcome their initial hesitations and fears. This involvement gradually fostered a sense of competence and trust, which aligns with the principles of FCC, known to reduce parental stress and improve neonatal outcomes [12]. Empowerment in this context was not just emotional but also functional, as mothers became more confident in understanding and interpreting their baby's developmental signals.

Observing tangible improvements in their neonates, such as better posture, more spontaneous movements, and improved breathing, further reinforced this sense of empowerment. Many mothers interpreted these positive changes as direct results of the physiotherapy and positioning interventions, which enhanced their trust in the care process. These perceived improvements not only offered hope but also contributed to a more optimistic view of their baby’s recovery. Some caregivers even linked these outcomes to earlier discharges, which further enhanced their sense of satisfaction and relief. These findings echo prior research that emphasizes how visible developmental progress can significantly impact caregiver well-being and reduce anxiety [13].

Beyond the NICU, caregivers reported increased readiness and confidence to continue supporting their infants’ care at home. This is a crucial outcome, as parental preparedness and reduced anxiety are known to influence long-term developmental outcomes and adherence to follow-up care. The involvement of caregivers in therapeutic practices thus creates a more sustainable care model that benefits both the child and family post discharge.

Importantly, this study draws attention to the dyadic relationship between the infant's physiological needs and the caregiver’s emotional state. In NICU settings, where the risk of psychological distress among caregivers is high, integrating family members into the therapeutic process not only supports the infant's physical development but also enhances caregiver's mental health. This holistic approach to neonatal care supports a growing consensus that emotional and developmental health are interdependent and must be addressed together [14].

Despite the strengths of this study, several limitations must be acknowledged. This study has some important limitations. Due to the sensitive NICU setting, interviews were kept short, which may have limited the depth of responses. The structured nature of the interview guide may have influenced participants’ answers, and the involvement of the authors in developing the novel device raises concerns about potential bias, which was not fully explored. While a large sample helped capture varied perspectives, it may have come at the cost of deeper individual insights. Additionally, direct participant quotes were not included in the final write-up, limiting transparency in how themes were developed. Future studies should aim for more open-ended interviews, include reflexivity, and present participant voices more clearly.

Future research should consider longitudinal designs to explore how early caregiver involvement in physiotherapy and positioning interventions affects long-term developmental and emotional outcomes. Multicenter studies could also enhance generalizability and identify context-specific factors that influence caregiver experiences. Additionally, integrating standardized tools to assess caregiver anxiety, confidence, and bonding could complement qualitative insights and provide a more comprehensive evaluation.

## Conclusions

This study highlights the profound and multifaceted experiences of mothers and caregivers of neonates who received physiotherapy interventions alongside novel positioning techniques in the NICU. In the analysis, caregivers reported significant emotional, functional, and participatory changes, emphasizing not only improvements in neonatal outcomes but also enhanced caregiver confidence, engagement, and readiness for post-discharge care. The findings enhance the importance of family-centered physiotherapy and positioning strategies in the NICU for preterm care.
